# Supplementary material for: Deep learning-based prediction of atrial fibrillation from polar transformed time-frequency electrocardiogram
Source: PLoS One. 2025 Mar 10;20(3):e0317630. doi: 10.1371/journal.pone.0317630 (PMC11892834; doi:10.1371/journal.pone.0317630)
Supplement: Attachment — Submitted filename: Response_to_reviewers 2024_0806.docx [file pone.0317630.s001.docx]

**Manuscript ID:** **PONE-D-24-23244R1**

**Title:** Deep learning-based prediction of atrial fibrillation from polar transformed time-frequency electrocardiogram

**Response to Review**

We thank the editor and reviewer for giving us the opportunity to revise this manuscript. Following the comments by the editor and reviewer, we have made significant changes to the manuscript. Listed below are responses to the comments along with descriptions of all changes to the manuscript.

**Journal requirements:**

1. Please ensure that your manuscript meets PLOS ONE's style requirements, including those for file naming. The PLOS ONE style templates can be found at https://journals.plos.org/plosone/s/file?id=wjVg/PLOSOne_formatting_sample_main_body.pdf and https://journals.plos.org/plosone/s/file?id=ba62/PLOSOne_formatting_sample_title_authors_affiliations.pdf

**Authors’ response:** As requested, we have modified our paper format to meet the style requirements.

2. Please note that PLOS ONE has specific guidelines on code sharing for submissions in which author-generated code underpins the findings in the manuscript. In these cases, we expect all author-generated code to be made available without restrictions upon publication of the work. Please review our guidelines at <https://journals.plos.org/plosone/s/materials-and-software-sharing#loc-sharing-code> and ensure that your code is shared in a way that follows best practice and facilitates reproducibility and reuse.

**Authors’ response:** We have shared our code to facilitate reproducibility in research. Our lab website at https://sites.google.com/yonsei.ac.kr/yoonckim/research/dl-prediction-of-afib-from-polar-transformed-ecg-spectrogram provides the Github links for the code.

3. We note that the grant information you provided in the ‘Funding Information’ and ‘Financial Disclosure’ sections do not match.

**Authors’ response:** We cannot modify the ‘Financial Disclosure’ section in the online submission site. Our Funding Statement should read as follows:

“This study was supported by the “Regional Innovation Strategy (RIS)” through the National Research Foundation of Korea (NRF) and funded by the Ministry of Education (MOE) (2022RIS-005).”

4. Thank you for stating the following in the Acknowledgments Section of your manuscript:

“This study was supported by the “Regional Innovation Strategy (RIS)” through the National Research Foundation of Korea (NRF) and funded by the Ministry of Education (MOE) (2022RIS-005).”

We note that you have provided funding information that is not currently declared in your Funding Statement. However, funding information should not appear in the Acknowledgments section or other areas of your manuscript. We will only publish funding information present in the Funding Statement section of the online submission form.

Please remove any funding-related text from the manuscript and let us know how you would like to update your Funding Statement. Currently, your Funding Statement reads as follows:

“The author(s) received no specific funding for this work.”

Please include your amended statements within your cover letter; we will change the online submission form on your behalf.

**Authors’ response:** We have removed the Acknowledgments section from the revised manuscript. Our Funding Statement should read as follows:

“This study was supported by the “Regional Innovation Strategy (RIS)” through the National Research Foundation of Korea (NRF) and funded by the Ministry of Education (MOE) (2022RIS-005).”

We have included our funding statement in our cover letter as well.

5. When completing the data availability statement of the submission form, you indicated that you will make your data available on acceptance. We strongly recommend all authors decide on a data sharing plan before acceptance, as the process can be lengthy and hold up publication timelines. Please note that, though access restrictions are acceptable now, your entire data will need to be made freely accessible if your manuscript is accepted for publication. This policy applies to all data except where public deposition would breach compliance with the protocol approved by your research ethics board. If you are unable to adhere to our open data policy, please kindly revise your statement to explain your reasoning and we will seek the editor's input on an exemption. Please be assured that, once you have provided your new statement, the assessment of your exemption will not hold up the peer review process.

**Authors’ response:** We have made the data available at our lab website at https://sites.google.com/yonsei.ac.kr/yoonckim/research/dl-prediction-of-afib-from-polar-transformed-ecg-spectrogram.

6. Please amend either the abstract on the online submission form (via Edit Submission) or the abstract in the manuscript so that they are identical.

**Authors’ response:** Thank you for pointing out the discrepancy. We ensured that they are identical in the revised manuscript submission.

**Additional Editor Comments:**

Reviewer agree that the manuscript contains novel elements. However, it presents some aspects that need to be solved before reconsideration.

1) The authors should explicitly mention the significant contributions of the manuscript. The novelty of the paper is not highlighted.

**Authors’ response:** We have provided the main contributions explicitly in the Introduction section of the revised manuscript.

[Introduction section, page 5]

The main contributions of this study can be summarized as follows.

1. A novel reverse polar transformed visual representation of time-frequency ECG spectrogram is presented and demonstrated for the identification of Afib in single lead ECG data.

2. The polar transformed ECG spectrogram images are used as input to a deep CNN model for the prediction of cardiac arrhythmia.

3. The effectiveness of the Pan-Tompkins (P-T) pre-processing algorithm is demonstrated for the prediction of cardiac arrhythmia when using deep CNNs.

2) The advantages and limitations of the proposed approach in relation to similar schemes are not clear.

**Authors’ response:** We have added the advantages and limitations to the Conclusions section.

[Conclusions section, page 16]

In sum, the proposed method is advantageous over the standard rectangular 2D representation with regard to its compact visualization of the long duration of ECG signal and its simplicity in implementation due to its square matrix form, which is suitable for existing and widely used 2D CNN models. The drawback of the proposed method in relation to the 1D ECG waveform analysis is that the color representation of the polar spectrogram is sensitive to the preprocessing filter and ECG signal amplitude.

3) Please revise the structure of the paper. It is recommendable to add to each section a couple of sentences that explain the purpose of the section. With this organization, the reader can clearly understand the sequence of the paper.

**Authors’ response:** We have added to each of the Methods and Results sections a couple of sentences that explain the purpose of the section.

[2. Methods section, page 5]

This section describes the ECG data used for our study, details of ECG signal pre-processing and polar transformation, and deep CNN model development and validation processes. The flowchart of the presented work is illustrated in Fig 1. ECG signals are processed to generate time-frequency spectrograms. After the polar coordinate transformation and mapping of it to the Cartesian grid, polar spectrogram images are generated, and then they are input to a deep CNN classifier model.

[2.4. Model development section, page 8]

This subsection describes the details of deep learning model development.

[3. Results section, page 11]

This section presents qualitative comparisons between polar transformed images and our proposed reverse polar transformed images. It also compares the visualization results between raw ECG signals and the P-T processed ECG signals. Quantitative results of deep CNN predictions on test data are shown across different CNN pre-trained models. The interpretation of deep CNN’s prediction results is made based on the reverse polar transformed spectrogram images.

4) Under the review observations, the paper should be corrected as a major revision.

**Authors’ response:** We sincerely thank the Editor for the decision.

**Reviewer comments:**

**Reviewer #1:**

This paper presents Deep learning-based prediction of atrial fibrillation. In general work is interesting although following issues are need to be resolve before ensuring recommendation.

**Authors’ response:** We greatly thank the reviewer for positive comments on our manuscript.

**R1.1:** The Research work is interesting but lags in terms of proving novelty in work.

**Authors’ response:** The novelty is in the demonstration of a reverse polar spectrogram representation of a long duration of ECG signal and its application to the deep CNN-based prediction of atrial fibrillation.

**R1.2:** Authors are required to write their contribution explicitly over the existing method. It seems that author has just used some methods and compared.

**Authors’ response:** We have provided the main contributions explicitly in the Introduction section of the revised manuscript.

[Introduction section, page 5]

The main contributions of this study can be summarized as follows.

1. A novel reverse polar transformed visual representation of time-frequency ECG spectrogram is presented and demonstrated for the identification of Afib in single lead ECG data.

2. The polar transformed ECG spectrogram images are used as input to a deep CNN model for the prediction of cardiac arrhythmia.

3. The effectiveness of the Pan-Tompkins (P-T) pre-processing algorithm is demonstrated for the prediction of cardiac arrhythmia when using deep CNNs.

**R1.3:** A little more mathematical analysis is required to support the proposed method.

**Authors’ response:** We have provided the mathematical description of the proposed transformation method in section 2.3. ‘Polar transformation’ of the revised manuscript on page 8 and 9.

**R1.4:** References are need to be formed and updated properly as recently developed methods are not included in the literature work.

**Authors’ response:** We have added references that include recently developed methods. This is related to the response to R1.5.

**R1.5:** In this work, following recently published publications can be added.

• Pal, H. S., Kumar, A., Vishwakarma, A., & Singh, G. K. (2023). Optimized Tunable-Q Wavelet Transform-Based 2-D ECG Compression Technique Using DCT. IEEE Transactions on Instrumentation and Measurement, 72, 1-13.

• Pal, H. S., Kumar, A., Vishwakarma, A., & Lee, H. N. (2023). Electrocardiogram signal compression using adaptive tunable-Q wavelet transform and modified dead-zone quantizer. ISA transactions, 142, 335-346.

• Pal, H. S., Kumar, A., Vishwakarma, A., Singh, G. K., & Lee, H. N. (2024). A new automated compression technique for 2D electrocardiogram signals using discrete wavelet transform. Engineering Applications of Artificial Intelligence, 133, 108123.

• Pal, H. S., Kumar, A., Vishwakarma, A., Singh, G. K., & Lee, H. N. (2024). An effective ECG signal compression algorithm with self controlled reconstruction quality. Computer Methods in Biomechanics and Biomedical Engineering, 27(7), 849-859.

• Gupta, K., Bajaj, V., & Jain, S. (2024). Multi-resolution assessment of ECG sensor data for sleep apnea detection using wide neural network. IEEE Sensors Journal.

• Gupta, K., Bajaj, V., & Ansari, I. A. (2023). Integrated s-transform-based learning system for detection of arrhythmic fetus. IEEE Transactions on Instrumentation and Measurement, 72, 1-8.

**Authors’ response:** We thank the reviewer for suggesting the references. We thought the suggested references are relevant to our work, and we cited them in the revised manuscript.
