# Supplementary material for: Deep learning-based prediction of atrial fibrillation from polar transformed time-frequency electrocardiogram
Source: PLoS One. 2025 Mar 10;20(3):e0317630. doi: 10.1371/journal.pone.0317630 (PMC11892834; doi:10.1371/journal.pone.0317630)
Supplement: Attachment — Submitted filename: Response_to_reviewers 2024_1202.docx [file pone.0317630.s002.docx]

**Manuscript ID:** **PONE-D-24-23244R2**

**Title:** Deep learning-based prediction of atrial fibrillation from polar transformed time-frequency electrocardiogram

**Response to Review**

We thank the editor and reviewers for giving us the opportunity to revise this manuscript. Following the comments by the editor and reviewer, we have made significant changes to the manuscript. Listed below are responses to the comments along with descriptions of all changes to the manuscript.

**Additional Editor Comments:**

The review has been received, and it indicates that major revisions are required. Please refer to the reviewer comments for specific areas needing improvement.

**Authors’ response:** We thank the editor for providing us with the opportunity to revise our manuscript.

**Reviewers’ comments:**

**Reviewer #1:**

All the comments are addressed.

**Authors’ response:** We greatly thank the reviewer for the positive comment on our manuscript.

**R1.1:** Improve the quality of figure 1-3 as fonts are not visible.

**Authors’ response:** We thank the reviewer for the comment. For improved visibility, we increased font size in Figures 1, 2, and 3 in the revised manuscript.

**R1.2:** Update the references as per the journal guidelines.

**Authors’ response:** We updated the references according to the guideline.

**R1.3:** Some grammatical mistakes need to be corrected, thus requires a thorough revision.

**Authors’ response:** We have reviewed the manuscript to check any grammatical errors after the major revision.

**Reviewer #2:**

**R2.1:** Discuss the motivation behind reverse polar transformed visual representation of time-frequency ECG spectrogram.

**Authors’ response:** We provided discussion on the motivation in the Introduction as follows.

[Introduction section, page 4, 2^nd^ paragraph]

The motivation behind the use of polar transformation stems from the intuition that for the case of a long duration of ECG signal, conventional spectrograms appear long in the horizontal axis, and the polar representation can provide compact visualization in a square form. Unlike iris-spectrograms, which are generated from one-heartbeat ECG signals [25], our method transforms longer duration ECG signals (30 seconds) into reverse polar spectrograms. Our approach to reverse polar transformation leverages the observation that high energy in ECG spectrograms resides in the low-frequency range. By reversing the frequency axis, high-energy regions are moved toward the periphery of the polar image, enhancing the visual discrimination between Afib and normal sinus rhythm.

**R2.2:** Deep learning models composed of the deep layers. By passing the ECG signal as an input directly to CNN model can predict or classify atrial fibrillation. For more details, authors can look at the article <https://link.springer.com/chapter/10.1007/978-3-319-68385-0_18>

**Authors’ response:** We thank the reviewer for providing us with the valuable reference. We cited the paper in our revised manuscript.

[Introduction section, page 3, 2^nd^ paragraph]

Deep learning models, including recurrent neural networks (RNNs), long short-term memory (LSTM), and gated recurrent unit (GRU) networks, have been utilized to identify Afib directly from raw ECG signals without requiring extensive preprocessing [14-16].

**R2.3:** The information behind transforming signal to an image is unclear.

**Authors’ response:** We clarified the information by rewriting the 2.2 Preprocessing of ECG signals and 2.3 Polar transformation sections.

**R2.4:** This database is not a new one and there are many related works on CNN and other deep learning models. The proposed method results should be compared with at least existing 3 methods.

**Authors’ response:** In the revised manuscript, we provided Table 3 to compare our proposed method with five selected existing methods, which utilized the CinC challenge 2017 data for evaluation.

**R2.5:** Discuss what are the advantages of the proposed method compared to the existing methods. Discuss the limitations of the proposed method.

**Authors’ response:** We summarized the advantages and limitations of the proposed method in the Conclusions section as follows.

[Conclusions section, page 24, last paragraph]

The proposed method offers advantages over traditional rectangular 2D spectrograms, such as compactly representing long-duration ECG signals and simplifying implementation due to its square matrix format, which is well-suited for widely used 2D CNN models. One limitation of the polar-transformed spectrogram is its sensitivity to preprocessing filters and ECG signal amplitude, which can affect the color representation. Nevertheless, this approach holds promise for improving ECG signal abnormality detection and is particularly well-suited to Afib detection with existing popular deep CNN classifiers.

**R2.6:** Hidden layer feature visualization can be shown for example penultimate layer feature visualization using t-SNE and add discussion on this. Feature also can be visualized using SHAP models. Discuss on these will support why the proposed model achieves better performances compared to the existing models.

**Authors’ response:** We provided the t-SNE feature visualization results in Figure 5. We did not consider SHAP models since we think they are more suited to tabular data analysis. We added discussion on the t-SNE results as follows.

[Results section, page 18]

Fig 5 presents t-Distributed Stochastic Neighbor Embedding (t-SNE) [44] visualizations of the penultimate feature distributions. Averaged penultimate features from the five cross-validated models were used as input to TSNE’s fit_transform() function in Scikit-Learn [45]. The distributions indicate that in well-performing models (e.g., MobileNet with P-T preprocessing in Fig 5d), intra-class samples are more tightly clustered compared to less effective models (e.g., ResNet50 without P-T preprocessing in Fig 5b).
